# Product-specific COVID-19 vaccine effectiveness against secondary infection in close contacts, Navarre, Spain, April to August 2021

**DOI:** 10.2807/1560-7917.ES.2021.26.39.2100894

**Published:** 2021-09-30

**Authors:** Iván Martínez-Baz, Camino Trobajo-Sanmartín, Ana Miqueleiz, Marcela Guevara, Miguel Fernández-Huerta, Cristina Burgui, Itziar Casado, María Eugenia Portillo, Ana Navascués, Carmen Ezpeleta, Jesús Castilla, the Working Group for the Study of COVID-19 in Navarre, Investigators, other members of the Working Group for the Study of COVID-19 in Navarre, Investigators, other members of the Working Group for the Study of COVID-19 in Navarre, Carmen Martín, Isabel Polo, Amaya Bacaicoa, Judith Chamorro, Juana Vidán, Ingrid Estévez, Igberto Tordoya, Delia Quílez, Francisco Lameiro, Ana Isabel Álvaro, Esther Albéniz, Aitziber Echeverría, Paula López Moreno, Javier Gorricho, Eva Ardanaz, Maite Arriazu, Fernando Baigorria, Aurelio Barricarte, Enrique de la Cruz, Jorge Díaz, Nerea Egüés, Manuel García Cenoz, Nerea Iriarte, Kenya Nekotxea, Conchi Moreno-Iribas, Carmen Sayón, Maitane Tellechea, Marian Nuín

**Affiliations:** 1 Instituto de Salud Pública de Navarra, Pamplona, Spain; 2 CIBER Epidemiología y Salud Pública (CIBERESP), Spain; 3 Navarra Institute for Health Research (IdiSNA), Pamplona, Spain; 4 Clinical Microbiology Department, Complejo Hospitalario de Navarra, Pamplona, Spain; 5 Other members of the Working Group for the Study of COVID-19 in Navarre are listed in the Investigators tab

**Keywords:** COVID-19, SARS-CoV-2, COVID-19 vaccine, cohort study, vaccine effectiveness, close contact

## Abstract

COVID-19 vaccine effectiveness by product (two doses Comirnaty, Spikevax or Vaxzevria and one of Janssen), against infection ranged from 50% (95% CI: 42 to 57) for Janssen to 86% (70 to 93) for Vaxzevria-Comirnaty combination; among ≥ 60 year-olds, from 17% (−26 to 45) for Janssen to 68% (48 to 80) for Spikevax; and against hospitalisation from 74% (43 to 88) for Janssen to > 90% for other products. Two doses of vaccine were highly effective against hospitalisation, but suboptimal for infection control.

Up to April 2021, four coronavirus disease (COVID-19) vaccines had been authorised by the European Medicines Agency to prevent severe acute respiratory syndrome coronavirus 2 (SARS-CoV-2) infections and to reduce the impact of this pandemic on hospital admissions and mortality. Comirnaty (BNT162b2 mRNA, BioNTech-Pfizer, Mainz, Germany/New York, United States (US)) and Spikevax (mRNA-1273, Moderna, Cambridge, US) are based on mRNA, while Vaxzevria (ChAdOx1 nCoV-19, Oxford-AstraZeneca, Cambridge, United Kingdom) and Janssen vaccine (Ad26.COV2-S, Janssen-Cilag International NV, Beerse, Belgium) are adenovirus-based vector vaccines [1-4]. Two doses are needed for full vaccination for all vaccines except Janssen, which only requires one dose.

We aimed to assess the product-specific COVID-19 vaccine effectiveness (VE) in preventing infection and hospitalisation in a prospective dynamic cohort of adults (≥ 18 years old) who were close contacts of COVID-19 cases from April to August 2021 in Navarre, Spain. We compared products in population groups that were vaccinated indistinctly with various products.

## Identification of close contacts and information sources

In Spain, to control COVID-19 transmission, all laboratory-confirmed COVID-19 cases are interviewed to detect their close contacts [5]. Close contacts are defined as any person who either had high-risk exposure to a confirmed COVID-19 case within a timeframe ranging from 2 days before the onset of symptoms in the case to 10 days after the onset of symptoms, or, for asymptomatic cases, in the 2 days before the sample which led to confirmation was taken, to 10 days after the sample was taken [6]. Close contacts are encouraged to be tested immediately and 7 to 10 days after the last exposure by RT-PCR. In symptomatic contacts, a positive antigen test within 5 days from the symptom onset is also considered a confirmed infection [7].

In our study, we excluded close contacts with a prior positive test, nursing home residents and those who did not complete the testing protocol. Age, sex, chronic conditions and contact setting were obtained from the enhanced epidemiological surveillance of COVID-19 [5]. Additional information about methods is described in the Supplementary Material.

## Vaccine products administered

In Spain, the COVID-19 vaccination campaign targeted adults starting with older age groups and gradually moving towards younger ones [8]. Although the product administered mainly depended on the availability at the particular moment, people aged ≥ 70 years were mainly vaccinated with Comirnaty or Spikevax, and those aged 60–69 years were mainly vaccinated with Vaxzevria. All products were used in people from 18 to 59 years of age; however, those who received the first dose of Vaxzevria could choose the second dose between Vaxzevria or Comirnaty. With this exception, all people received the same product for the second dose.

Information about COVID-19 vaccine doses, product and date of administration were obtained from the regional vaccination register. We considered a person fully vaccinated ≥ 14 days after receiving one dose of Janssen or the second dose of other vaccines, and partially vaccinated ≥ 14 days after receiving only the first dose of Spikevax, Comirnaty or Vaxzevria.

## Statistical analysis of product-specific vaccine effectiveness

We compared the incidence of SARS-CoV-2 infection, of symptomatic cases and of COVID-19 hospitalisation by product-specific COVID-19 vaccination status as a variable with nine categories, with unvaccinated people as reference category. The same risk period was assigned to everyone in the cohort; therefore, the Cox regression provided estimates of the crude and adjusted relative risks (RR) with 95% confidence intervals (CI). Adjusted models included age groups, sex, chronic conditions, contact setting, month and COVID-19 vaccination status of the index case.

The VE was estimated as a percentage: (1 − adjusted RR) x 100. Product-specific effectiveness was estimated in close contacts by age group (18–59 and ≥ 60 years), contact setting (household or other), vaccination status of the index case and by SARS-CoV-2 variant (Phylogenetic Assignment of Named Global Outbreak (Pango) lineage designation B.1.1.7 (Alpha), B.1.617.2 (Delta) and other). For each product and number of doses, infection incidence ≥ 90 days after the last dose was compared to infection incidence within 90 days after the last dose. As individuals aged 18–59 years could have been vaccinated with all products, the relative VE in this age group was used to compare vaccine products in an analysis limited to 90 days from the last dose to reduce product differences in time since the last dose.

## Estimates of product-specific vaccine effectiveness

The study cohort included 30,240 close contacts of 12,263 index cases. In the cohort of close contacts, 7,177 (23.7%) SARS-CoV-2 infections were confirmed and 263 (3.7%) of them led to hospitalisation. Characteristics of the study population by outcomes and product-specific vaccination status are shown in Table 1 and Supplementary Tables S1 and S2.

**Table 1:** COVID-19 outcomes by characteristics of close contacts and vaccination status of their index cases, dynamic cohort^a^, Navarre, Spain, April–August 2021 (n = 30,240)

Characteristics	Total	All SARS-CoV-2 infections^b^		Symptomatic SARS-CoV-2 infections		COVID-19 hospital admission	
		Number	Secondary attack rate, %	Number	Secondary attack rate, %	Number	Secondary attack rate, %
Total	30,240	7,177	24	4,676	16	263	0.9
Age groups (years)							
18–34	9,608	2,929	31	1,839	19	21	0.2
35–49	7,655	1,810	24	1,278	17	60	0.8
50–69	10,286	1,893	18	1,220	12	119	1.2
≥ 70	2,691	545	20	339	13	63	2.3
Sex							
Male	14,590	3,553	24	2,182	15	138	0.9
Female	15,650	3,624	23	2,494	16	125	0.8
Major chronic conditions							
No	21,706	5,214	24	3,413	16	137	0.6
Yes	8,534	1,963	23	1,263	15	126	1.5
Vaccination status of close contacts							
Unvaccinated	14,348	4,811	34	3,278	23	216	1.5
Partially vaccinated^c^	4,138	723	18	405	10	16	0.4
Fully vaccinated^c^	11,754	1,643	14	993	8	31	0.3
Contact setting							
Household	16,305	4,639	29	3,096	19	212	1.3
Other	13,935	2,538	18	1,580	11	51	0.4
Month of contact (2021)							
April	7,137	2,143	30	1,550	22	155	2.2
May	2,988	722	24	527	18	35	1.2
June	2,077	457	22	332	16	7	0.3
July	13,138	2,817	21	1,531	12	38	0.3
August	4,900	1,038	21	736	15	28	0.6
Vaccination status of index case							
Unvaccinated	25,024	6,237	25	4,078	16	236	0.9
Partially vaccinated ^c^	1,729	328	19	202	12	12	0.7
Fully vaccinated ^c^	3,487	612	18	396	11	15	0.4

COVID-19: coronavirus disease; SARS-CoV-2: severe acute respiratory syndrome coronavirus 2.

^a^ Close contacts with a prior positive test, nursing home residents and those who did not complete the testing protocol were excluded.

^b^ All infections included asymptomatic and symptomatic SARS-CoV-2 infections.

^c^ A person was considered fully vaccinated ≥ 14 days after receiving one dose of Janssen or the second dose of other vaccines, and partially vaccinated ≥ 14 days after receiving only the first dose of Spikevax, Comirnaty or Vaxzevria.

The adjusted point VE estimate against COVID-19 hospitalisation was higher than 90% for two vaccine doses of any product, and was 74% (95% CI: 43 to 88) for one dose of Janssen. Effectiveness of full vaccination in preventing SARS-CoV-2 infection ranged from 50% (95% CI: 42 to 57) for Janssen to 86% (95% CI: 70 to 93) for the Vaxzevria-Comirnaty combination. The VE of full vaccination with two doses was 82% (95% CI: 78 to 86) for Spikevax, 69% (95% CI: 66 to 72) for Comirnaty and 54% (95% CI: 48 to 60) for Vaxzevria. The VE estimates were similar or higher against symptomatic infection (Table 2).

**Table 2:** Product-specific COVID-19 vaccine effectiveness, dynamic cohort, Navarre, Spain, April–August 2021 (n = 30,240)

COVID-19 outcome evaluated and vaccination status	Cases / total	Secondary attack rate, %	Crude VE (95% CI)	Adjusted VE (95% CI)^b^
All SARS-CoV-2 infections^a^				
Unvaccinated	4,811/14,348	34	Reference	Reference
1 dose of Janssen	209/997	21	37 (28 to 46)	50 (42 to 57)
1 dose of Spikevax	70/517	14	60 (49 to 68)	66 (56 to 73)
2 doses of Spikevax	85/1,127	8	77 (72 to 82)	82 (78 to 86)
1 dose of Comirnaty	351/2,022	17	48 (42 to 53)	57 (52 to 61)
2 doses of Comirnaty	1,070/7,972	13	60 (57 to 62)	69 (66 to 72)
1 dose of Vaxzevria	302/1,599	19	44 (37 to 50)	41 (34 to 48)
2 doses of Vaxzevria	272/1,539	18	47 (40 to 53)	54 (48 to 60)
1 dose of Vaxzevria + 1 dose of Comirnaty	7/119	6	82 (63 to 92)	86 (70 to 93)
Symptomatic SARS-CoV-2 infection				
Unvaccinated	3,278/14,348	23	Reference	Reference
1 dose of Janssen	126/997	13	45 (34 to 54)	54 (45 to 62)
1 dose of Spikevax	38/517	7	68 (56 to 77)	71 (61 to 79)
2 doses of Spikevax	46/1,127	4	82 (76 to 87)	85 (80 to 89)
1 dose of Comirnaty	182/2,022	9	61 (54 to 66)	66 (60 to 71)
2 doses of Comirnaty	645/7,972	8	65 (61 to 67)	72 (69 to 75)
1 dose of Vaxzevria	185/1,599	12	49 (41 to 56)	46 (37 to 54)
2 doses of Vaxzevria	173/1,539	11	51 (43 to 58)	56 (48 to 63)
1 dose of Vaxzevria + 1 dose of Comirnaty	3/119	3	89 (66 to 96)	91 (71 to 97)
COVID-19 hospital admission				
Unvaccinated	216/14,348	1.5	Reference	Reference
1 dose of Janssen	8/997	0.8	47 (−8 to 74)	74 (43 to 88)
1 dose of Spikevax	2/517	0.4	74 (−3 to 94)	73 (−10 to 93)
2 doses of Spikevax	1/1,127	0.1	96 (58 to 99)	98 (82 to 100)
1 dose of Comirnaty	6/2,022	0.3	80 (56 to 92)	86 (69 to 94)
2 doses of Comirnaty	20/7,972	0.3	83 (74 to 89)	93 (88 to 96)
1 dose of Vaxzevria	8/1,599	0.5	67 (33 to 84)	78 (54 to 89)
2 doses of Vaxzevria	2/1,539	0.1	91 (65 to 98)	95 (79 to 99)
1 dose of Vaxzevria + 1 dose of Comirnaty	0/119	0	100 (NA)	100 (NA)

CI: confidence interval; COVID-19: coronavirus disease; NA: not applicable; SARS-CoV-2: severe acute respiratory syndrome coronavirus 2; VE: vaccine effectiveness.

^a^ Cases included asymptomatic and symptomatic SARS-CoV-2 infections.

^b^ VE adjusted by age group (18–34, 35–49, 50–69 and ≥ 70 years), sex, major chronic conditions, contact setting (household or other), month and vaccination status of index case.

These analyses estimate the VE by product, but product comparison may not be valid.

The effectiveness of full vaccination in preventing SARS-CoV-2 infection was higher in people aged 18–59 years than in those aged ≥ 60 years for all vaccine products. Among people aged ≥ 60 years, full vaccination estimates ranged from 17% (95% CI: − 26 to 45) for Janssen to 68% (95% CI: 48 to 80) for Spikevax (Table 3).

**Table 3:** Product-specific COVID-19 vaccine effectiveness against SARS-CoV-2 infection by age group, dynamic cohort, Navarre, Spain, April–August 2021 (n = 30,240)

Study population and vaccination status	Cases/total	Secondary attack rate, %	Crude VE (95% CI)	Adjusted VE (95% CI)^a^
Close contacts 18–59 years old				
Unvaccinated	4,386/13,114	33	Reference	Reference
1 dose of Janssen	180/892	20	40 (30 to 48)	52 (44 to 59)
1 dose of Spikevax	63/489	13	61 (51 to 70)	68 (58 to 75)
2 doses of Spikevax	65/912	7	79 (73 to 83)	83 (78 to 86)
1 dose of Comirnaty	291/1,738	17	50 (44 to 55)	60 (54 to 64)
2 doses of Comirnaty	743/5,863	13	62 (59 to 65)	70 (67 to 73)
1 dose of Vaxzevria	100/588	17	49 (38 to 58)	45 (33 to 55)
2 doses of Vaxzevria	148/808	18	45 (35 to 53)	54 (46 to 61)
1 dose of Vaxzevria +1 dose of Comirnaty	7/119	6	82 (63 to 92)	86 (70 to 93)
Close contacts ≥ 60 years old				
Unvaccinated	425/1,234	34	Reference	Reference
1 dose of Janssen	29/105	28	20 (−17 to 45)	17 (−26 to 45)
1 dose of Spikevax	7/28	25	27 (−53 to 66)	25 (–59 to 65)
2 doses of Spikevax	20/215	9	73 (58 to 83)	68 (48 to 80)
1 dose of Comirnaty	60/284	21	39 (20 to 53)	32 (8 to 49)
2 doses of Comirnaty	327/2,109	16	55 (48 to 61)	50 (37 to 60)
1 dose of Vaxzevria	202/1,011	20	42 (31 to 51)	24 (3 to 40)
2 doses of Vaxzevria	124/731	17	51 (40 to 60)	44 (25 to 57)

CI: confidence interval; COVID-19: coronavirus disease; SARS-CoV-2: severe acute respiratory syndrome coronavirus 2; VE: vaccine effectiveness.

^a^ VE adjusted by age group (18–34, 35–49, 50–69 and ≥ 70 years), sex, major chronic conditions, contact setting (household or other), month and vaccination status of index case.

These analyses estimate the VE by product, but product comparison may not be valid.

The VE estimates did not change substantially when close contacts of vaccinated index cases were excluded or only household contacts were considered (Table 4).

**Table 4:** Product-specific COVID-19 vaccine effectiveness against SARS-CoV-2 infection by vaccination status of the index case and contact setting, dynamic cohort, Navarre, Spain, April–August 2021 (n = 30,240)

Study population and close contact vaccination status	Cases/total	Secondary attack rate, %	Crude VE (95% CI)	Adjusted VE (95% CI)^a^
Close contacts of unvaccinated index cases				
Unvaccinated	4,559/13,485	34	Reference	Reference
1 dose of Janssen	151/779	19	43 (33 to 51)	54 (46 to 62)
1 dose of Spikevax	55/393	14	59 (46 to 68)	65 (54 to 73)
2 doses of Spikevax	55/850	6	81 (75 to 85)	85 (80 to 88)
1 dose of Comirnaty	272/1,569	17	49 (42 to 55)	57 (51 to 62)
2 doses of Comirnaty	716/5,606	13	62 (59 to 65)	70 (67 to 73)
1 dose of Vaxzevria	246/1,277	19	43 (35 to 50)	42 (33 to 49)
2 doses of Vaxzevria	176/982	18	47 (38 to 54)	55 (47 to 62)
1 dose of Vaxzevria + 1 dose of Comirnaty	7/83	8	75 (48 to 88)	80 (59 to 91)
Close contacts of fully-vaccinated index cases				
Unvaccinated	118/394	30	Reference	Reference
1 dose of Janssen	47/167	28	6 (−32 to 33)	23 (−14 to 48)
1 dose of Spikevax	8/77	10	65 (29 to 83)	64 (26 to 83)
2 doses of Spikevax	24/220	11	64 (43 to 76)	70 (52 to 81)
1 dose of Comirnaty	38/216	18	41 (15 to 59)	43 (18 to 61)
2 doses of Comirnaty	286/1,839	16	48 (36 to 58)	59 (45 to 69)
1 dose of Vaxzevria	18/124	15	51 (20 to 70)	43 (2 to 67)
2 doses of Vaxzevria	73/420	17	42 (22 to 57)	41 (16 to 58)
1 dose of Vaxzevria + 1 dose of Comirnaty	0/30	NA	NA	NA
Household close contacts				
Unvaccinated	2,869/6,494	44	Reference	Reference
1 dose of Janssen	179/741	24	45 (36 to 53)	42 (32 to 51)
1 dose of Spikevax	42/254	17	63 (49 to 72)	62 (49 to 72)
2 doses of Spikevax	68/769	9	80 (74 to 84)	79 (73 to 84)
1 dose of Comirnaty	248/1,152	22	51 (44 to 57)	51 (44 to 58)
2 doses of Comirnaty	811/5,048	16	64 (61 to 66)	65 (62 to 69)
1 dose of Vaxzevria	232/913	25	42 (34 to 50)	35 (25 to 43)
2 doses of Vaxzevria	185/863	21	51 (44 to 58)	50 (41 to 58)
1 dose of Vaxzevria + 1 dose of Comirnaty	5/71	7	84 (62 to 93)	84 (61 to 93)
Non-household close contacts				
Unvaccinated	1,942/7,854	25	Reference	Reference
1 dose of Janssen	30/256	12	53 (32 to 67)	54 (33 to 68)
1 dose of Spikevax	28/263	11	57 (37 to 70)	66 (50 to 76)
2 doses of Spikevax	17/358	5	81 (69 to 88)	83 (72 to 90)
1 dose of Comirnaty	103/870	12	52 (42 to 61)	56 (46 to 64)
2 doses of Comirnaty	259/2,924	9	64 (59 to 68)	68 (62 to 73)
1 dose of Vaxzevria	70/686	10	59 (48 to 67)	45 (29 to 57)
2 doses of Vaxzevria	87/676	13	48 (35 to 58)	54 (42 to 63)
1 dose of Vaxzevria + 1 dose of Comirnaty	2/48	4	83 (33 to 96)	86 (43 to 96)

CI: confidence interval; COVID-19: coronavirus disease; NA: not applicable; SARS-CoV-2: severe acute respiratory syndrome coronavirus 2; VE: vaccine effectiveness.

^a^ VE adjusted by age group (18–34, 35–49, 50–69 and ≥ 70 years), sex, major chronic conditions, contact setting (household or other), month and vaccination status of index case.

These analyses estimate the vaccine effectiveness by product, but product comparison may not be valid.

The time since the last dose was < 90 days for most vaccinated close contacts. The VE declined 90 days after the second dose of Spikevax (85% to 67%; p_comparison_ = 0.003) and Comirnaty (70% to 63%; p_comparison_ = 0.035), but not 90 days after the first dose of Vaxzevria (40% to 52%; p_comparison_ = 0.746) (Supplementary Table S3).

Several VE estimates seemed slightly lower against the Delta variant than against the Alpha variant; however, all products maintained a notable protective effect against both variants (Supplementary Table S4).

### Comparisons of product-specific full vaccination effectiveness

Among people aged 18–59 years, product-specific VE within 90 days since the last dose was compared. Compared with vaccination with Janssen, the relative VE was 66% (95% CI: 54 to 75) for Spikevax, 39% (95% CI: 28 to 49) for Comirnaty and 69% (95% CI: 34 to 86) for the Vaxzevria-Comirnaty combination. Similarly, full vaccination with Spikevax (66%; 95%CI: 53 to 75), Comirnaty (38%; 95% CI: 26 to 48) and the Vaxzevria-Comirnaty combination (69%; 95% CI: 33 to 85) were more effective than two doses of Vaxzevria.

### Ethical statement

This study was approved by the Navarre’s Ethical Committee for Clinical Research (PI2020/45), which waived the requirement of obtaining informed consent.

## Discussion

The results show a clear effectiveness of the full COVID-19 vaccination with each of the four evaluated vaccine products and highlight some improvement points. Our findings are consistent with previous studies that reported high effectiveness of full COVID-19 vaccination in preventing hospitalisations [9-11] and moderate VE in preventing symptomatic and asymptomatic SARS-CoV-2 infection [12,13]. However, few studies evaluated VE for specific products [11,14].

Two doses of mRNA vaccine or the heterologous vector/mRNA vaccination provided high protection against infection in the population < 60 years of age. However, a decrease in the VE was observed from 90 days after the last dose that raises doubts about the duration of this effect.

The COVID-19 VE for the same product was lower in the elderly people. Therefore, full vaccination effect seemed suboptimal to prevent infection in this age group and to achieve transmission control in locations with a high proportion of older population as has been shown in other studies [10,12]. Younger age groups vaccinated with one dose of Janssen or two doses of Vaxzevria had also a suboptimal protection against SARS-CoV-2 infection [13].

Our results are consistent with the stronger immunological response observed in individuals who received the Vaxzevria-Comirnaty combination than those who received two doses of Vaxzevria [15,16]. This supports the recommendation of this heterologous vaccination in Spain and several other countries [8,17].

Although the COVID-19 VE may be slightly lower against the Alpha and Delta variants, full vaccination remains notably protective against these variants of concern as also shown by Lopez Bernal et al. [18].

Regardless of the product, two-dose vaccination remained highly effective in preventing hospitalisation; however, the VE of one dose of Janssen seemed slightly lower. The suboptimal VE in preventing infection in fully vaccinated people, because of older age, vaccine type or time from the last dose, may be insufficient for infection control. A booster dose of vaccine in some cases has been suggested as a possible solution [19,20].

The present study has advantages of the cohort design while all cases were laboratory-confirmed and non-cases were those who tested negative for SARS-CoV-2 as in the test-negative design and the analysis of close contacts ensures similar exposure to infection [12,13,21]. However, this study also has some limitations. Vaccination coverage varied with age, comorbidity and month; nevertheless, analyses were adjusted for these variables. Since characteristics of people who received each vaccine product were different, specific estimates can be considered separately and care must be taken in comparisons. The correlation among close contacts of the same index case was not controlled for. Epidemiological and vaccination conditions of this study may not be similar to other sites. Estimates refer to the first 8 months since vaccination and VE may wane over time.

### Conclusions

Regardless of the product, two vaccine doses were highly effective against hospitalisation, and mRNA or heterologous vaccination provided high protection within 90 days against SARS-CoV-2 infection in those younger than 60 years of age. However, protection against infection was suboptimal for vector-based vaccines in adults and for all products among people ≥ 60 years old. Additional preventive measures should be maintained while SARS-CoV-2 is circulating to interrupt transmission in the population and product-specific VE should be monitored in the long term.

## Supplementary Data

309000 Supplementary Material
