# Supplementary material for: Product-specific COVID-19 vaccine effectiveness against secondary infection in close contacts, Navarre, Spain, April to August 2021
Source: Euro Surveill. 2021 Sep 30;26(39):2100894. doi: 10.2807/1560-7917.ES.2021.26.39.2100894 (PMC8485582; doi:10.2807/1560-7917.ES.2021.26.39.2100894)
Supplement: Supplementary Material [file 21-00894_CATALAN_Supplementary_material.pdf]

SUPPLEMENTARY MATERIAL

This supplementary material is hosted by *Eurosurveillance* as supporting information alongside the article "Product-specific COVID-19 vaccine effectiveness against secondary infection in close contacts, Navarre, Spain, April to August 2021" on behalf of the authors who remain responsible for the accuracy and appropriateness of the content. The same standards for ethics, copyright, attributions and permissions as for the article apply. Supplements are not edited by *Eurosurveillance* and the journal is not responsible for the maintenance of any links or email addresses provided therein.

Supplementary Methods

As part of measures to control COVID-19, all laboratory-confirmed COVID-19 cases, according to the European Union definition [1], were interviewed to identify their close contacts [2]. A close contact of a COVID-19 case was defined as any person who had had high-risk exposure to a confirmed COVID-19 case within a timeframe ranging from 2 days before the onset of symptoms in the case to 10 days after the onset of symptoms, or in the 2 days before the sample, which led to confirmation was taken, to 10 days after the sample was taken for asymptomatic cases [3]. Close contacts were tested by reverse-transcription (RT)-PCR for SARS-CoV-2 in nasopharynx samples initially and 10 days after the last contact. In symptomatic contacts, a positive antigen test within 5 days from the symptom onset was also considered confirmatory of SARS-CoV-2 infection, because these tests have demonstrated very high specificity [4]. Close contacts with a positive test for SARS-CoV-2 before January 2021, nursing home residents and those who did not complete the testing protocol were excluded from the study. COVID-19 hospitalisation entailed admission due to laboratory-confirmed COVID-19 for ≥24 hours. Age, sex, chronic conditions, healthcare work and contact setting (household or other) were obtained from the enhanced epidemiological surveillance of COVID-19 [2].

Household contacts were those close contacts who lived in the same home at least one night during the infectiousness period of the index case.

This study analysed the cohort of adults who were close contact of COVID-19 cases from 1 April to 31 August 2021 in Navarre, Spain. The follow-up period to detect events in the close contacts lasted until 13 September 2021. Each contact was only included once in the present study.

Although one index case could have more than one close contact in the study, each close contact was considered as an independent observation, because the study aim was to evaluate the vaccine effectiveness in the close contacts and vaccination status and other characteristics could be very different even among close contacts of the same index case.

COVID-19 vaccine doses, product and date of administration were obtained from the regional vaccination register. We defined a person fully vaccinated ≥ 14 days after receiving one dose of Janssen or the second dose of other vaccines, and partially vaccinated ≥ 14 days after receiving the first dose of Spikevax, Comirnaty or Vaxzevria. Contacts who have received the first dose <14 days before testing were excluded from the analyses.

Index cases samples with RT-PCR cycle threshold ≤30 were tested by TaqPath COVID-19 RT-PCR kit and TaqMan SARS-CoV-2 Mutation Panel (Thermo Fisher Scientific, USA). S target–negative status by TaqPath was used as a proxy of the Alpha variant, and the L452R mutation by TaqMan for the Delta variant. Brand-specific effectiveness was estimated in close contacts by aged group (18-59 and ≥60 years), contact setting (household or other), vaccination status of the index cases and by probably SARS-CoV-2 variant (Phylogenetic Assignment of Named Global Outbreak (Pango) lineage designation B.1.1.7 (Alpha), B.1.617.2 (Delta) and other).

We compared the incidence of SARS-CoV-2 infection, of symptomatic cases and of COVID-19 hospitalisation by product-specific COVID-19 vaccination status as a variable with nine categories, with unvaccinated people as reference category. The same risk period was assigned to everyone in the cohort; therefore, the Cox regression provided estimates of the crude and adjusted relative risks (RR) with 95% confidence intervals (CI). Adjusted models included age groups, sex, chronic conditions, contact setting, month and COVID-19 vaccination status of the index case.

The VE was estimated as a percentage: (1 − adjusted RR) x 100. Product-specific effectiveness was estimated in close contacts by age group (18–59 and ≥ 60 years),

contact setting (household or other), vaccination status of the index case and by SARS-CoV-2 variant. For each product and number of doses, infection incidence ≥90 days after the last dose was compared to infection incidence within 90 days after the last dose. As individuals aged 18–59 years could have been vaccinated with all products, the relative VE in this age group was used to compare vaccine products in an analysis limited to 90 days from the last dose to reduce product differences in time since the last dose.

The dates of approval of each COVID-19 vaccine brand in Spain were:

- Comirnaty: 21 December 2020
- Spikevax: 6 January 2021
- Vaxzevria: 29 January 2021
- Janssen: 11 March 2021

References

1. European Center for Disease Control and Prevention (ECDC). Case definition for coronavirus disease 2019 (COVID-19, as of 3 December 2020. Stockholm: ECDC. Available from: https://www.ecdc.europa.eu/en/covid-19/surveillance/case-definition

2. Ministerio de Sanidad. Estrategia de detección precoz, vigilancia y control de COVID-19. Actualizado a 26 de febrero de 2021. [Strategy for early detection, surveillance and control of COVID-19. Updated 26 February 2021]. Madrid: Spanish Ministry of Health. Spanish. Available from: https://www.mscbs.gob.es/profesionales/saludPublica/ccayes/alertasActual/nCov/documentos/COVID19_Estrategia_vigilancia_y_control_e_indicadores.pdf

3. European Centre for Disease Prevention and Control (ECDC). Contact tracing: public health management of persons, including healthcare workers, who have had contact with COVID-19 cases in the European Union –third update, 18 November2020. Stockholm: ECDC; 2020. Available from: https://www.ecdc.europa.eu/sites/default/files/documents/covid-19-contact-tracing-public-health-management-third-update.pdf

4. European Centre for Disease Prevention and Control (ECDC). Options for the use of rapid antigen tests for COVID-19 in the EU/EEA and the UK. 19 November 2020. ECDC: Stockholm; 2020. Available from: https://www.ecdc.europa.eu/sites/default/files/documents/Options-use-of-rapid-antigen-tests-for-COVID-19_0.pdf

Supplementary Table S1. Characteristics of the study population of close contacts of COVID-19 index cases confirmed by brand-specific vaccination status of the close contacts**.** Navarre, Spain, April to August 2021 (n = 30,240)

	**Unvaccinated**		**Janssen 1 dose**		**Spikevax 1 dose**		**Spikevax 2 doses**		**Comirnaty 1 dose**		**Comirnaty 2 doses**		**Vaxzevria 1 dose**		**Vaxzevria 2 doses**		**Vaxzevria + Comirnaty**	
	**Number**	**%**	**Number**	**%**	**Number**	**%**	**Number**	**%**	**Number**	**%**	**Number**	**%**	**Number**	**%**	**Number**	**%**	**Number**	**%**
**Age groups (years)**																		
18–34	7,635	53.2	7	0.7	263	50.9	98	8.7	499	24.7	569	7.1	200	12.5	291	18.9	46	38.7
35–49	3,568	24.9	310	31.1	119	23.0	95	8.4	910	45.0	2,197	27.6	205	12.8	213	13.8	38	31.9
50–69	2,681	18.7	636	63.8	109	21.1	742	65.8	360	17.8	3,494	43.8	1,194	74.7	1,035	67.3	35	29.4
≥70	464	3.2	44	4.4	26	5.0	192	17.0	253	12.5	1,712	21.5	0	0.0	0	0.0	0	0.0
**Age, median (IQR)**	32 (22-48)		51 (48-55)		34 (24-52)		55 (53-58)		45 (35-51)		53 (47-63)		61 (49-63)		59 (44-62)		42 (25-50)	
**Sex**																		
Male	7,526	52.5	510	51.2	263	50.9	513	45.5	949	46.9	3,418	42.9	755	47.2	620	40.3	36	30.3
Female	6,822	47.5	487	48.8	254	49.1	614	54.5	1,073	53.1	4,554	57.1	844	52.8	919	59.7	83	69.7
**Major chronic conditions**																		
No	11,001	76.7	726	72.8	391	75.6	728	64.6	1,450	71.7	5,180	65.0	1,069	66.9	1,071	69.6	90	75.6
Yes	3,347	23.3	271	27.2	126	24.4	399	35.4	572	28.3	2,792	35.0	530	33.1	468	30.4	29	24.4
**Contact setting**																		
Household	6,494	45.3	741	74.3	254	49.1	769	68.2	1,152	57.0	5,048	63.3	913	57.1	863	56.1	71	59.7
Other	7,854	54.7	256	25.7	263	50.9	358	31.8	870	43.0	2,924	36.7	686	42.9	676	43.9	48	40.3
**Median time since the last dose (IQR)**	NA		54 (40-69)		25 (18-34)		41 (28-65)		25 (19-30)		50 (31-88)		68 (42-84)		34 (23-48)		41 (32-55)	
**Median time between the first and second dose (IQR)**	NA		NA		NA		27 (27-28)		NA		20 (20-21)		NA		83 (82-100)		99 (86-107)	
**Total**	**14,348**	**100**	**997**	**100**	**517**	**100**	**1,127**	**100**	**2,022**	**100**	**7,972**	**100**	**1,599**	**100**	**1,539**	**100**	**119**	**100**

IQR, interquartile range; NA, not applicable.

Supplementary Table S2. Characteristics of the study population of close contacts of COVID-19 index cases confirmed by brand-specific vaccination status of the close contacts**.** Navarre, Spain, April to August 2021 (n = 30,240)

	Unvaccinated		Janssen 1 dose		Spikevax 1 dose		Spikevax 2 doses		Comirnaty 1 dose		Comirnaty 2 doses		Vaxzevria 1 dose		Vaxzevria 2 doses		Vaxzevria + Comirnaty	
	**Number**	**%**	**Number**	**%**	**Number**	**%**	**Number**	**%**	**Number**	**%**	**Number**	**%**	**Number**	**%**	**Number**	**%**	**Number**	**%**
**Month of contact**																		
April	6,231	43.4	3	0.3	20	3.9	12	1.1	141	7.0	353	4.4	377	23.6	0	0.0	0	0.0
May	2,225	15.5	12	1.2	19	3.7	25	2.2	161	8.0	251	3.1	295	18.4	0	0.0	0	0.0
June	1,172	8.2	61	6.1	60	11.6	51	4.5	173	8.6	312	3.9	216	13.5	28	1.8	4	3.4
July	4,061	28.3	656	65.8	271	52.4	757	67.2	1,156	57.2	4,505	56.5	667	41.7	978	63.5	87	73.1
August	659	4.6	265	26.6	147	28.4	282	25.0	391	19.3	2,551	32.0	44	2.8	533	34.6	28	23.5
**Vaccination status of index case**																		
Unvaccinated	13,485	94.0	779	78.1	393	76.0	850	75.4	1,569	77.6	5,606	70.3	1,277	79.9	982	63.8	83	69.7
Partially vaccinated	469	3.3	51	5.1	47	9.1	57	5.1	237	11.7	527	6.6	198	12.4	137	8.9	6	5.0
Fully vaccinated	394	2.7	167	16.8	77	14.9	220	19.5	216	10.7	1,839	23.1	124	7.8	420	27.3	30	25.2
**Total**	14,348	100	997	100	517	100	1,127	100	2,022	100	7,972	100	1,599	100	1,539	100	119	100

IQR, interquartile range; NA, not applicable.

Supplementary Table S3. Brand-specific COVID-19 vaccine effectiveness against SARS-CoV-2 infection by time since the last dose. Navarre, Spain, April to August 2021 (n = 30,240)

**Vaccination status**	**<90 days since last dose vs unvaccinated**			**≥90 days since last dose vs unvaccinated**		
	**Cases/ total**	**Crude VE (95% CI)**	**Adjusted VE (95% CI)** [a]	**Cases/ total**	**Crude VE (95% CI)**	**Adjusted VE (95% CI)** [a]
Unvaccinated	4,811/14,348	Reference	Reference	4,811/14,348	Reference	Reference
1 dose of Janssen	185/926	40 (31 to 49)	52 (44 to 59)	24/71	−1 (−51 to 32)	28 (−8 to 53)
1 dose of Spikevax	70/516	59 (49 to 68)	65 (56 to 73)	0/1	NA	NA
2 doses of Spikevax	62/973	81 (76 to 85)	85 (80 to 88)	23/154	55 (33 to 70)	67 (50 to 78) [b]
1 dose of Comirnaty	351/2,011	48 (42 to 53)	57 (51 to 61)	0/11	NA	NA
2 doses of Comirnaty	761/6,032	62 (59 to 65)	70 (67 to 73)	309/1,940	52 (47 to 58)	63 (58 to 68) [b]
1 dose of Vaxzevria	251/1,325	43 (36 to 50)	40 (31 to 47)	51/274	44 (27 to 58)	52 (37 to 64) [b]
2 doses of Vaxzevria	272/1,536	47 (40 to 53)	54 (47 to 60)	0/3	NA	NA
1 dose of Vaxzevria + 1 dose of Comirnaty	7/117	82 (63 to 91)	85 (69 to 93)	0/2	NA	NA

CI: confidence interval; VE: vaccine effectiveness; NA: not applicable.
[a] VE adjusted by age group (18-34, 35-49, 50-69 and ≥70 years), sex, major chronic conditions, contact setting (household or other), month and vaccination status of index case.
[b] Comparison of the incidence of infection ≥ 90 days after the last dose of vaccine versus the incidence of infection within 90 days after the last dose of vaccine: for the second dose of Spikevax 67% vs 85%, p = 0.003; for the second dose of Comirnaty 63% vs 70%, p = 0.035; and for the first dose of Vaxzevria 52% vs 40%, p = 0.746.
These analyses estimate the vaccine effectiveness by product, but product comparison may not be valid.

Supplementary Table S4. Brand-specific COVID-19 vaccine effectiveness against all infections by SARS-CoV-2 variants. Navarre, Spain, April to August 2021

SARS-CoV-2 variant and vaccination status	Cases/ total	Crude VE (95% CI)	Adjusted VE (95% CI) [a]
**Alpha variant**			
Unvaccinated	1,116/3,977	Reference	Reference
1 dose of Janssen	3/35	69 (5 to 90)	77 (27 to 93)
1 dose of Spikevax	7/55	55 (5 to 78)	60 (14 to 81)
2 doses of Spikevax	3/65	84 (49 to 95)	86 (56 to 95)
1 dose of Comirnaty	43/276	44 (25 to 59)	54 (37 to 67)
2 doses of Comirnaty	58/557	63 (52 to 71)	71 (61 to 78)
1 dose of Vaxzevria	89/488	35 (19 to 48)	37 (21 to 50)
2 doses of Vaxzevria	6/30	29 (–59 to 68)	38 (–42 to 73)
1 dose of Vaxzevria + 1 dose of Comirnaty	0/0	NA	NA
**Delta variant**			
Unvaccinated	460/990	Reference	Reference
1 dose of Janssen	45/213	54 (38 to 66)	42 (18 to 59)
1 dose of Spikevax	12/98	74 (53 to 85)	72 (51 to 84)
2 doses of Spikevax	22/232	80 (69 to 87)	77 (64 to 85)
1 dose of Comirnaty	56/357	66 (55 to 74)	63 (51 to 73)
2 doses of Comirnaty	242/1,759	70 (65 to 75)	67 (59 to 74)
1 dose of Vaxzevria	24/155	67 (50 to 78)	53 (26 to 70)
2 doses of Vaxzevria	61/385	66 (55 to 74)	55 (39 to 67)
1 dose of Vaxzevria + 1 dose of Comirnaty	2/32	86 (46 to 97)	86 (45 to 97)
**Non-Alpha and non-Delta variant**			
Unvaccinated	159/494	Reference	Reference
1 dose of Janssen	8/38	35 (–33 to 68)	37 (–39 to 71)
1 dose of Spikevax	2/19	67 (–32 to 92)	65 (–46 to 92)
2 doses of Spikevax	0/26	NA	NA
1 dose of Comirnaty	9/75	63 (27 to 81)	67 (32 to 84)
2 doses of Comirnaty	26/180	55 (32 to 70)	58 (25 to 76)
1 dose of Vaxzevria	9/60	53 (9 to 76)	39 (–29 to 71)
2 doses of Vaxzevria	4/27	54 (–24 to 83)	58 (–19 to 85)
1 dose of Vaxzevria + 1 dose of Comirnaty	0/1	NA	NA

CI: confidence interval; VE: vaccine effectiveness; NA: not applicable.
[a] VE adjusted by age group (18-34, 35-49, 50-69 and ≥70 years), sex, major chronic conditions, contact setting (household or other), month and vaccination status of index case.
These analyses estimate the vaccine effectiveness by product, but product comparison may not be valid.
